# Navy Medical Appointments

**Published:** 1859-07

**Authors:** 


NAVY MEDICAL APPOINTMENTS.—The Naval Medical Board of Examiners, consisting of Surgeons W. S. W. Ruschenberger, L. B. Hunter, T. D. Miller, and Passed Assistant Surgeon G. H. Howell, adjourned in this city, on the twenty-third of May, having recommended the following gentlemen for admission to the post of Assistant Surgeons in the Navy:—1. William Bradley, of Pennsylvania; 2. Edw. F. Corson, of Pennsylvania; 3. David Kindleberger, of Ohio; 4. T. D. Grafton, of Arkansas; 5. R. L. Weber, of Pennsylvania; 6. R. J. Freeman, of Virginia; 7. W. E. Taylor, of Virginia; 8. B. W. Green, of Virginia; 9. T. McMaster, of Pennsylvania; 10. T. W. Herty, of Georgia.

Assistant Surgeons T. J. Turner, W. G. Hay, R. P. Daniel, and W. S. Hood, were examined by the Board, and having acquitted themselves satisfactorily, were recommended for promotion.

